# Gene Correlation Guided Gene Selection for Microarray Data Classification

**DOI:** 10.1155/2021/6490118

**Published:** 2021-08-14

**Authors:** Dong Yang, Xuchang Zhu

**Affiliations:** ^1^Department of Colorectal Surgery, Tianjin Union Medical Center, Tianjin 300121, China; ^2^Department of Gastrointestinal Surgery, Lianshui People's Hospital Affiliated to Kangda College of Nanjing Medical University, Huai'an 223300, China

## Abstract

The microarray cancer data obtained by DNA microarray technology play an important role for cancer prevention, diagnosis, and treatment. However, predicting the different types of tumors is a challenging task since the sample size in microarray data is often small but the dimensionality is very high. Gene selection, which is an effective means, is aimed at mitigating the curse of dimensionality problem and can boost the classification accuracy of microarray data. However, many of previous gene selection methods focus on model design, but neglect the correlation between different genes. In this paper, we introduce a novel unsupervised gene selection method by taking the gene correlation into consideration, named gene correlation guided gene selection (G^3^CS). Specifically, we calculate the covariance of different gene dimension pairs and embed it into our unsupervised gene selection model to regularize the gene selection coefficient matrix. In such a manner, redundant genes can be effectively excluded. In addition, we utilize a matrix factorization term to exploit the cluster structure of original microarray data to assist the learning process. We design an iterative updating algorithm with convergence guarantee to solve the resultant optimization problem. Experimental results on six publicly available microarray datasets are conducted to validate the efficacy of our proposed method.

## 1. Introduction

During cell division and growth, abnormal changes often happen to genes, which results in varying cancers. With the rapid development of kinds of biomedical technologies [1], DNA microarray comes into being and lots of microarray data can be obtained for cancer prevention, diagnosis, and treatment [2–12]. For various microarray data, classifying the different types of tumors is an important task, but challenging due to the high dimensionality and small numbers of samples [13–15] since the small number of data samples with large number of genes can easily result in the “curse of dimensionality” and overfitting problems of data processing and learning models. When the dimension of samples is too high, the distance between any two samples is very inaccurate. Therefore, the classification task for this kind of data is often challenging. However, it has been verified by some existing biological experiments that only a very small proportion of genes contribute significantly to biological process and disease indication. Directly processing original high dimensional microarray data not only degenerates the classification performance but also brings extra computation burden of hardware. Therefore, it is necessary to select a subset of discriminative genes from high-dimensional microarray data to serve subsequent tasks [16–25]. If we treat each gene as a feature dimension in microarray data, gene selection is similar to the feature selection task in machine learning and data mining community [26–37]. In fact, many feature selection methods can be used well for gene selection. Therefore, mathematical gene selection methods can be also grouped into three classes, i.e., filter methods, wrapper methods, and embedded methods.

Filter methods often measure the importance of different genes in a straight-forward manner based on certain criteria such as *t*-test [38, 39], *Z*-score [40], signal-to-noise ratio (SNR) [41], Laplacian score [42], mutual information [43], and information gain [44]. In [41], Golub et al. firstly used the SNR function to evaluate the weights of the genes. Many traditional feature selection methods such as ReliefF [45] and MRMR [46] are combined and used for gene selection [47]. Since filter methods only depend on the intrinsic properties of original data [48], a good ranking criterion function is very important.

As to wrapper methods, varying classification algorithms are often used as a fitness evaluation to determine the subset of genes and the selected genes can in turn enhance the classification performance [2, 49–56]. In general, wrapper methods can obtain better results than filter methods, but bring more expensive computational cost. A lot of evolutionary algorithms such as genetic algorithm (GA), differential evolution (DE), ant colony optimization (ACO), and simulated annealing are commonly used as wrapper methods for gene selection [57, 58].

For embedded methods, the geometric structure and intrinsic property of data are exploited to construct gene selection models. Among this kind of methods, some mathematical regularization terms with specific physic meanings such as representative and sparse characters are commonly used assumptions. Typical models include self-representation [32, 33, 59–62], low-rank representation [63, 64], and matrix factorization [65–67]. Based on these basic models, many variants have been proposed, such as Laplacian graph regularized low-rank representation [63]. Considering the robustness to outliers, Wang et al. [66] proposed a robust *l*_2,1_-norm regularized characteristic gene selection method. In [68], Guo et al. proposed to identify the disease-associated genes by utilizing ensemble consensus-guided unsupervised feature selection method. In an unsupervised manner, the major priori information can be used is the intrinsic local geometric structure of data. Therefore, embedded methods that use this priori information can achieve good performance for various of microarray data and obtain more and more attention.

**Figure 1:**
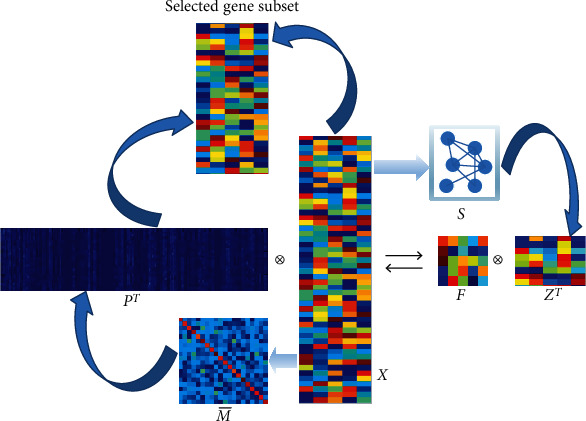
Brief flowchart of our proposed G^3^CS model.

Although there are lots of computational methods proposed for gene selection and achieve great success, most of them focus on the relation of data samples while the correlation between different genes is ignored. The expression values of different genes should be interrelated for a certain microarray data matrix. Therefore, we propose to calculate the correlation of gene pairs to regularize the gene selection model, which is named as named gene correlation guided gene selection (G^3^CS). In detail, in order to exclude redundant genes, the covariance of different gene dimension pairs is calculated and embedded into our unsupervised gene selection model to regularize the gene selection coefficient matrix. In addition, we utilize a matrix factorization model which can capture the cluster structure of original data to assist the learning process. We design an iterative updating algorithm to solve the resultant problem. Finally, experimental results on six publicly available real microarray datasets are conducted to demonstrate that the proposed G^3^CS can steadily perform better than other state-of-the-art computational gene selection methods in terms of microarray data classification. In Figure 1, we give a brief flowchart of our proposed G^3^CS model.

## 2. Related Work

In this section, we introduce some gene selection works that are most related to our proposed method. Before that, we firstly present some notations will be used in the following sections. Throughout this paper, matrices and vectors are denoted as boldface capital letters boldface lower case letters, respectively. Given an matrix **X** ∈ ℝ^*m*×*n*^, **X**_*ij*_ represents its (*i*, *j*)-th element, **x**^*i*^ and **x**_*j*_ denotes its *i*-th row and *j*-th column, respectively. **X**^*T*^ is the transpose of **X**. If **X** is square, *Tr*(**X**) is the trace of **X**. **I**_*k*_ denotes an identity matrix with size *k* × *k*. 1 is a vector with all elements are 1. ${\left\|X\right\|}_{2,1}=\sum_{i=1}^{m}\left\|x^{i}\right\|=\sum_{i=1}^{m}\sqrt{\sum_{j=1}^{n}X_{ij}^{2}}$ denotes the *l*_2,1_-norm of matrix **X**, which is used to constrain the row sparsity of **X**. ${\left\|X\right\|}_{F}=\sqrt{\sum_{i=1}^{n}\sum_{j=1}^{m}X_{ij}^{2}}$ is the well-known Frobenius norm of **X**.

Since our proposed G^3^CS belongs to the embedded method, we give a brief review about some related embedded methods.

### 2.1. GRSL-GS

In [20], Tang et al. proposed a manifold regularized subspace learning model for gene selection, in which the model projects original high dimensional microarray data into a lower-dimensional subspace, then original genes are constrained to be well represented by the selected gene subset. In order to capture the local manifold structure of original data, a Laplacian graph regularization term is imposed on the low-dimensional data space. Finally, the learned projection matrix can be regarded as an important indicator of different genes. Specifically, the mathematical model of GRSL-GS can be formulated as follows:

(1) $$\begin{array}{c} \begin{array}{c} \underset{C,P}{\arg\min}{\left\|X-XPC\right\|}_{F}^{2}+\lambda Tr\left(P^{T}X^{T}LXP\right)\\ \text{s}.\text{t}.P\geq 0,C\geq 0,P^{T}P=I, \end{array} \end{array}$$

where **P** denotes the projection matrix, **C** represents the data reconstruction coefficient matrix, and **L** is the Laplacian matrix calculated from original data. *λ* is a hyper-parameter that balances the two regularization terms. The first term in Eq. (1) constraints that original microarray data can be reconstructed from the projected lower-dimensional gene dictionary, and the second term is the graph Laplacian regularization term used to preserve the intrinsic local manifold structure of original data samples. Although GRSL-GS captures the local structure information, it does not exploit the gene correlation.

### 2.2. AHEDL

Considering that the graph Laplacian in GRSL-GS can only capture pairwise sample relationship, Zheng et al. [22] introduced a computational gene selection model via adaptive hypergraph embedded dictionary learning (AHEDL). Similar to GRSL-GS, AHEDL also learns a dictionary from original high dimensional microarray data, and the learned dictionary is then used to represent original data by a reconstruction coefficient matrix. The difference of dictionary learning between GRSL-GS and AHEDL is that GRSL-GS uses projection process but AHEDL directly utilizes traditional dictionary learning model. The *l*_2,1_-norm is imposed on the coefficient matrix for selecting discriminate genes.

In addition, the hypergraph is also learned in an adaptive manner. In a nutshell, AHEDL can be formulated as follows:

(2) $$\begin{array}{c} \begin{array}{c} \underset{D,C,H_{e},H_{v},W}{\min}{\left\|X-DC\right\|}_{F}^{2}+\alpha Tr\left({CLC}^{T}\right)+\beta {\left\|C\right\|}_{2,1}+\gamma Tr\left(W^{T}W\right)\\ \text{s}.\text{t}.{\left\|d_{i}\right\|}^{2}\leq 1,w^{T}1=1,w\left(e_{i}\right)>0, \end{array} \end{array}$$

As can be seen from Eq. (2), AHEDL integrates adaptive hypergraph learning, dictionary learning, and gene selection into a uniform framework. The dictionary matrix **D**, representation coefficient matrix **C** and hypergraph **W** can constrain each other during the optimization process to obtain their optimums. Since **D** can be regarded as the new representation of **X** in the dictionary space, the row sparsity imposed on **C** by using the *l*_2,1_-norm can be used to measure the importance of gene dimensions in the learned dictionary space.

## 3. Proposed Method

Given a microarray data **X** ∈ **R**^*m*×*n*^, which contains *n* data samples with *m* different genes. Gene selection aims to select a gene subset that contains only a small number of genes for subsequent tasks. Without sample label information, we should exploit the intrinsic structure of data as much as possible. In this work, we deploy traditional regression model as the basic architecture to formulate G^3^CS, which can be represented as follows:

(3) $$\begin{array}{c} \underset{P}{\min}{\left\|P^{T}X-C\right\|}_{F}^{2}+\alpha {\left\|P\right\|}_{2,1}, \end{array}$$

where **P** ∈ R^*m*×*c*^ is a projection matrix that projects original data into label space **C** = [**c**_1_, ⋯, **c**_*n*_] ∈ {0, 1}^*c*×*n*^, where **c**_*i*_ ∈ {0, 1}^*c*^ is the cluster indicator vector corresponding to **x**_*i*_. In order to measure the importance of different genes, we impose the *l*_2,1_-norm on **P** to constrain that important genes contribute more during the projection process. In machine learning and data mining community, matrix factorization of target matrix **C** often shows remarkable performance [67, 69]. In our G^3^CS model, we also decompose **C** into two components, i.e., **F** ∈ *R*^*c*×*c*^ and **Z** ∈ *R*^*n*×*c*^. As a result, Eq. (3) can be rewritten as following form with appropriate constraints:

(4) $$\begin{array}{c} \begin{array}{c} \underset{P,F,Z}{\min}{\left\|P^{T}X-{FZ}^{T}\right\|}_{F}^{2}+\alpha {\left\|P\right\|}_{2,1}\\ \text{s}.\text{t}.F^{T}F=I,Z^{T}Z=I,Z\geq 0, \end{array} \end{array}$$

where **F**^*T*^**F** = **I** constrain each column of **B** to be independent with each other. **Z**^*T*^**Z** = **I** is a relaxation constraint that makes each row of **Z** to have only one nonzero element. The constraints in Eq. (8) make the model to conduct orthogonal clustering which works well for unsupervised feature selection [70].However, by minimizing Eq. (8) directly for gene selection neglects the gene correlation information which is important in biomedical process. In this work, we embed the gene correlation information into G^3^CS. It is well known that in probability theory and statistics, a covariance matrix is a square matrix giving the covariance between each pair of elements of a given random vector. In this work, we use covariance to calculate the correlation of different gene pairs, then, we can get a symmetric semipositive definite covariance matrix **M**. The *i*, *j*-th entry of covariance matrix **M** can be calculated as follows:

(5) $$\begin{array}{c} M\left(i,j\right)=\frac{\sum_{i=1}^{m}\left(x^{i}-\bar{x}\right)\left(x^{i}-\bar{x}\right)}{m-1}, \end{array}$$

where $\bar{x}$ is the gene average vector, which is calculated as follows:

(6) $$\begin{array}{c} \bar{x}=\frac{\sum_{i=1}^{m}{\text{x}}^{i}}{m}. \end{array}$$

However, the diagonal elements in **M** only reflect the relationship between a gene dimension and itself, which makes no sense in our model. Therefore, we adjust **M** to get a new correlation matrix $\bar{M}$ by the following equation:

(7) $$\begin{array}{c} \bar{M}\left(i,j\right)=\left\{\begin{array}{cc} M\left(i,j\right) & \text{if }i=j,\\ \underset{k\neq i}{\sum }M\left(i,k\right) & \text{if }i\neq j. \end{array}\right. \end{array}$$

In such a manner, $\bar{M}\left(i,j\right)$ represents the correlation between the *i*-th gene dimension with all other gene dimensions. Then, $\bar{M}\left(i,j\right)$ can be embedded into Eq. (8) to emphasize the independence of selected gene dimensions from the perspective of data information. Therefore, we have

(8) $$\begin{array}{c} \underset{P,\;F,Z}{\min}{\left\|P^{T}X-{FZ}^{T}\right\|}_{F}^{2}+\alpha {\left\|P\right\|}_{2,1}+\beta Tr\left(P^{T}\bar{M}P\right),\\ \text{s}.\text{t}.F^{T}F=I,Z^{T}Z=I,Z\geq 0. \end{array}$$

In addition, the local geometric structure information of original data should be preserved as much as possible in the learned new space **Z**. By using the Gaussian kernel function, we can get a similarity matrix from original data by the following equation:

(9) $$\begin{array}{c} S_{ij}=\left\{\begin{array}{cc} \exp\left(\frac{{\left\|x_{i}-x_{j}\right\|}^{2}}{-2t^{2}}\right), & x_{i}\in N_{k}\left(x_{j}\right)\text{ or }x_{j}\in N_{k}\left(x_{i}\right);\\ 0, & \text{otherwise}, \end{array}\right. \end{array}$$

where *𝒩*_*k*_(**x**_*i*_) represents the set of *k* nearest neighbors of **x**_*i*_, and *t* is a width parameter. *k* and *t* are set to 5 and 0.5, respectively, in our experiments. In our G^3^CS model, we require that if two data samples are closed to each other in original space, their cluster indicator vectors in new space **Z** should also be close. This constraint can be formulated by using the following form:

(10) $$\begin{array}{c} \underset{z}{\min}\frac{1}{2}\overset{n}{\underset{i=1}{\sum }}\overset{n}{\underset{j=1}{\sum }}{\left\|z^{i}-z^{j}\right\|}_{2}^{2}S\left(i,j\right)=\underset{z}{\min}Tr\left(Z^{T}LZ\right), \end{array}$$

where **L** is the Laplacian matrix corresponding to **S**. Finally, we get the mathematical formulation of our G^3^CS model as follows:

(11) $$\begin{array}{c} \underset{P,F,Z}{\min}{\left\|P^{T}X-{FZ}^{T}\right\|}_{F}^{2}+\alpha {\left\|P\right\|}_{2,1}+\beta Tr\left(P^{T}\bar{M}P\right)+\gamma Tr\left(Z^{T}LZ\right)\\ \text{s}.\text{t}.F^{T}F=I,Z^{T}Z=I,Z\geq 0. \end{array}$$

where *α*, *β*, and *γ* are three hyperparameters to balance different regularization terms. In summary, Eq. (1) integrates regression, matrix factorization, gene correlation, and data local structure exploitation into a unified framework. The gene correlation regularizes the model to exclude redundant gene dimensions.

## 4. Optimization Algorithm

There are three variables in Eq. (1) that need to be optimized; we cannot obtain a close-form solution simultaneously for all of them. Therefore, we design an algorithm to iteratively update these variables. For each time, we update a variable by fixing other ones.

### 4.1. Optimize *P*

When other variables are fixed, solving **P** is equal to the following problem:

(12) $$\begin{array}{c} \underset{P}{\min}{\left\|P^{T}X-{FZ}^{T}\right\|}_{F}^{2}+\alpha {\left\|P\right\|}_{2,1}+\beta Tr\left(P^{T}\bar{M}P\right). \end{array}$$

By taking the derivative of Eq. (12) with respect to **P** and setting it to zero, we have

(13) $$\begin{array}{c} 2XX^{T}P-2XZF^{T}+2\alpha GP+2\beta \bar{M}P=0, \end{array}$$

Then, we have the closed-form solution of **P** as follows:

(14) $$\begin{array}{c} P={\left(XX^{T}+\alpha G+\beta \bar{M}\right)}^{-1}XZF^{T}, \end{array}$$

where **G** is a diagonal matrix with **G**_*ii*_ = 1/2‖P^*i*^‖_2_. At each iteration, **G** and **P** can be updated alternatively.

### 4.2. Optimize **F**

When fixing other variables, the optimization problem is equal to the following equation:

(15) $$\begin{array}{c} \underset{F}{\min}{\left\|P^{T}X-FZ^{T}\right\|}_{F}^{2}\;s.t.F^{T}F=I. \end{array}$$

By adding a constant matrix **Z** into the *F*-norm, Eq. (15) is equal to

(16) $$\begin{array}{c} \underset{F}{\min}{\left\|\left(P^{T}X-FZ^{T}\right)Z\right\|}_{F}^{2}s.t.F^{T}F=I. \end{array}$$

Since **Z** is an orthogonal matrix, then, we have

(17) $$\begin{array}{c} \underset{F}{\min}{\left\|W-F\right\|}_{F}^{2}s.t.F^{T}F=I. \end{array}$$

where **W** = **P**^*T*^**XZ**. In order to ensure the orthogonal constraint of **F**, we add a large positive constant *ρ* and the optimization problem can be converted to

(18) $$\begin{array}{c} \underset{F}{\min}\frac{1}{2}{\left\|W-F\right\|}_{F}^{2}+\frac{\rho }{4}{\left\|F^{T}F-I\right\|}_{F}^{2}. \end{array}$$

By setting the derivative of Eq. (18) respect to **F** to 0, we have

(19) $$\begin{array}{c} -W+F+\rho \left(FF^{T}F-F\right)=0, \end{array}$$

then **F** can be updated by the following equation in each iteration:

(20) $$\begin{array}{c} F_{ij}=\frac{W_{ij}}{{\left[F+\rho \left({FF}^{T}F-F\right)\right]}_{ij}}. \end{array}$$

### 4.3. Optimize **Z**

When fixing other variables, the optimization problem for **Z** is equal to the following equation:

(21) $$\begin{array}{c} \underset{\text{Z}}{\min}{\left\|P^{T}X-{FZ}^{T}\right\|}_{F}^{2}+\gamma Tr\left(Z^{T}LZ\right)\\ \text{s}.\text{t}.Z^{T}Z=I,Z\geq 0. \end{array}$$

We add a penalty term for the constraint **Z**^*T*^**Z** = **I** and a Lagrange multiplier for the constraint **Z** ≥ 0. Then, the Lagrange function for Eq. (21) can be written as follows:

(22) $$\begin{array}{c} L\left(Z,\alpha \right)=\underset{Z}{\min}{\left\|P^{T}X-{FZ}^{T}\right\|}_{F}^{2}+\gamma Tr\left(Z^{T}LZ\right)+\frac{\kappa }{4}\left\|Z^{T}Z-I\right\|+Tr\left(\alpha Z^{T}\right). \end{array}$$

By setting the derivative of Eq. (22) with respect to **Z** to 0, we have

(23) $$\begin{array}{c} -2X^{T}PF+2ZF^{T}F+\kappa \left(ZZ^{T}Z-Z\right)+\alpha =0. \end{array}$$

According to the Kuhn-Tucker conditions *α*_*ij*_**Z**_*ij*_ = 0, we have

(24) $$\begin{array}{c} Z_{ij}=\frac{{\left[2X^{T}PF+\kappa Z\right]}_{ij}}{{\left[2ZF^{T}F+\kappa ZZ^{T}Z\right]}_{ij}}. \end{array}$$

After we solve the resultant optimization problem as described by Eq. (1), we can measure the importance of each gene dimension by calculating the *l*_2_-norm of each row of **P**. We summarize the optimization procedure of the G^3^CS model in Algorithm 1.

The proposed algorithm converges well with increasing iteration times. In our experiments, when the objective function value change between two continuous iteration times is very small, we stop the optimization process and obtain good results.

## 5. Experimental Results

In this section, extensive experiments are conducted on several real microarray datasets to validate the efficacy of the proposed G^3^CS. In order to demonstrate that the gene subset selected by G^3^CS can obtain better classification results, we use three kinds of classification algorithms including Support Vector Machine (SVM), Random Forest (RF), and *k*-nearest neighbor (KNN) to test the selected gene subset obtained by different previous gene selection methods.

### 5.1. Microarray Datasets

Six publicly available microarray datasets are used in our experiments, which are colon cancer (colon) [71], B-cell chronic lymphocytic leukemia (CLL SUB 111), breast, lung, tumors-11, and global cancer map (GCM) (1CLL SUB 111 and lung can be downloaded from: http://featureselection.asu.edu/datasets.php; breast and GCM can be downloaded from: http://portals.broadinstitute.org/cgi-bin/cancer/datasets.cgi; tumors-11 can be downloaded from: http://datam.i2r.a-star.edu.sg/datasets/krbd/index.html.) and are used to test the performance of the proposed G^3^CS and other gene selection methods used for comparison. These datasets are collected for diagnosis of different kinds of cancers such as colon cancer, lung cancer, Ewing's family of tumors, non-Hodgkin lymphoma, and rhabdomyosarcoma and prostate cancer. For an instance, CLL SUB 111 contains high-density oligonucleotide arrays which can be used to identify molecular correlates of genetically and clinically distinct subgroups of B-cell chronic lymphocytic leukemia (B-CLL). Lung is a dataset used to determine whether global biological differences underlie common pathological features of prostate cancer and to identify genes that might anticipate the clinical behaviour of this disease.

It should be noted that the above six datasets are typical with high-dimensional genes. In each dataset, the number of genes is much larger than the number of samples, which brings challenge for many practical tasks. In Table 1, we give a brief description about these datasets.

### 5.2. Experimental Settings

In the proposed G^3^CS, we have three parameters that need to be adjusted, i.e., *α*, *β*, and *γ*. In our experiments, we varied their values by a “grid-search” in the range {10^−3^, 10^−2^, 10^−1^, 1, 10, 10^2^, 10^3^}. In addition, the optimal number of selected genes is also unknown, we set different numbers of selected genes for different datasets, and the final best results obtained from the optimal parameter setting were reported. In our experiments, the number of selected genes was tuned from {10,20,30,40,50} for each dataset. For each gene subset, the three abovementioned different basic classification methods were used to classify the microarray data for testing the discrimination of selected genes. In order to validate the efficacy of the proposed G^3^CS, we compare it with other six state of-the-art gene selection methods including:

- *F*-test [72], which is a traditional filter-based gene selection method, it uses the statistical hypothesis testing
- RLR [73], which is based on linear discriminant analysis criterion. The class centroid is estimated to define both the between-class separability and the within-class compactness
- WLMGS (Weight Local Modularity based Gene Selection) [74], which uses the weight local modularity of a weighted sample graph to evaluate the discriminative power of gene subset
- LNNFW [75], which uses the *k*-nearest neighbors rule to minimize the within-class distances and maximize the between-class distances
- GRSL-GS [20], which is based on subspace learning and manifold regularization
- AHEDL [22], which is based on dictionary learning theory with adaptive hypergraph learning and regularization

As to WLMGS and GRSL-GS, we set the number of nearest neighbor for constructing the sample graph to 5. The kernel width *σ* used in the Gaussian kernel function and other regularization parameters in GRSL-GS and RLR are tuned with 5-fold cross validation (CV). For other parameters in other methods, we use the recommended settings in the corresponding references. We run all the implementation programs on a desktop computer with Intel Core i5-4200M 2.5 GHz CPU and 8 GB RAM.

### 5.3. Experimental Comparison of Different Methods

In order to verify the superiority of the proposed G^3^CS, we compare it with the other six state-of-the-art gene selection methods on different datasets. For each dataset, we can obtain 5 different gene subsets with the numbers of selected genes which vary from 10 to 50. As to each gene subset, three classifiers and 5-fold CV are used for classification performance evaluation, and we report the average accuracy of 5 times of CV in Table 2. We mark the best results in bold font for clear comparison. As can be seen from the results, the proposed G^3^CS can consistently outperform other methods in terms of averaged classification accuracy, which demonstrates that G^3^CS can effectively select more discriminative genes for original high-dimensional microarray data for classification task.

### 5.4. Classification Accuracy with Different Numbers of Selected Genes

**Figure 2:**
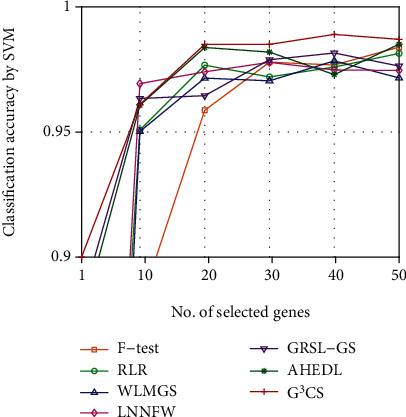
The classification accuracy of different methods with different selected number of genes on colon dataset.

**Figure 3:**
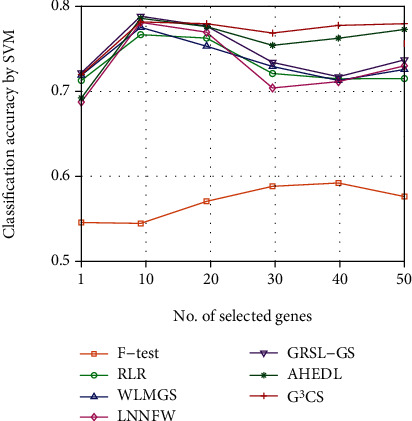
The classification accuracy of different methods with different selected number of genes on CLL SUB 111 dataset.

**Figure 4:**
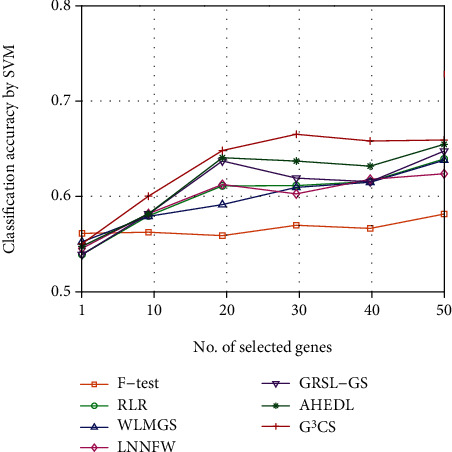
The classification accuracy of different methods with different selected number of genes on breast dataset.

**Figure 5:**
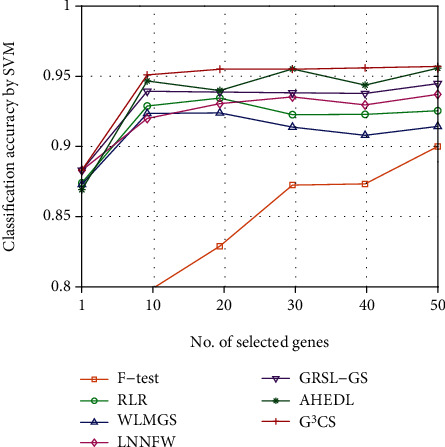
The classification accuracy of different methods with different selected number of genes on lung dataset.

**Figure 6:**
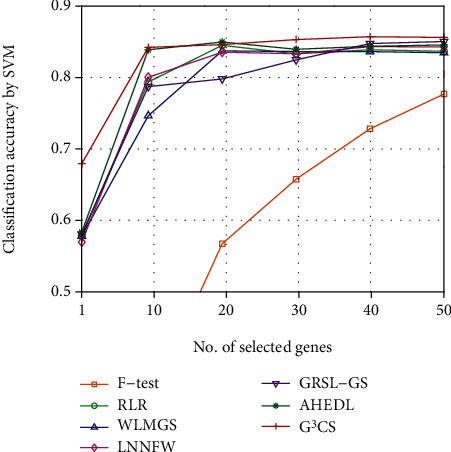
The classification accuracy of different methods with different selected number of genes on tumors-11 dataset.

**Figure 7:**
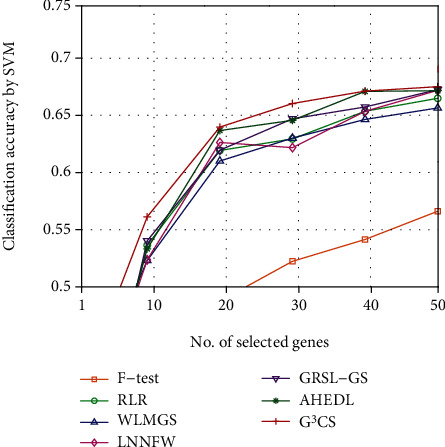
The classification accuracy of different methods with different selected number of genes on GCM dataset.

Since the optimal number of selected genes for each dataset is hard to determine, we investigate the classification performance of different methods on different datasets with different numbers of selected genes. We plot the classification accuracy curves of different methods on different datasets with varied numbers of selected genes in Figures 2–7. For each method and each dataset, we plot the average classification accuracy value of the 5 times CV obtained by the SVM classifier. As can be seen from Figures 2–7, the proposed G^3^CS performs steadily better than other methods when the number of selected genes changes. With a small number of selected genes, our method can select more discriminative genes than other methods, which validates that the selected gene subset obtained by G^3^CS can better serve classification of microarray data.

## 6. Discussion and Conclusions

In this work, we present a novel gene selection method by taking the gene correlation into consideration, named gene correlation guided gene selection (G^3^CS). In detail, we capture the correlation of different gene dimension pairs by calculating the covariance matrix from the perspective of gene dimension and embed it into the proposed model to regularize the gene selection coefficient learning. In such a manner, redundant genes can be effectively excluded to reduce the redundancy of the selected genes. In addition, a matrix factorization term was utilized to exploit the cluster structure of original microarray data to assist the learning process. We design an iterative updating algorithm to solve the resultant optimization problem. Experimental results on six publicly available microarray datasets are conducted to validate the efficacy of our proposed method. With varied selected gene dimensions, the proposed method can consistently outperform other compared ones in terms of classification accuracy.

## Figures and Tables

**Algorithm 1:**
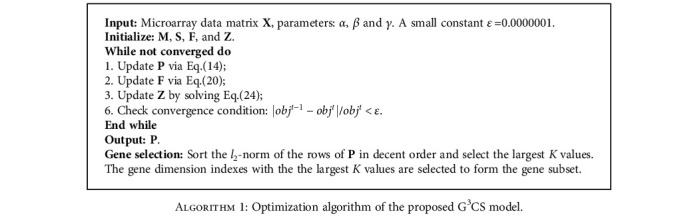
Optimization algorithm of the proposed G^3^CS model.

**Table 1:** Statistics of the microarray datasets used in our experiments.

Datasets	#instance	#gene number	#class
Colon	62	20000	2
Lung	203	12600	5
Tumors-11	174	12533	11
CLL_SUB_111	111	11340	3
Breast	95	4869	3
GCM	198	16063	14

**Table 2:** Averaged classification accuracy (ACC ± SD) of different methods by using different classifiers (%) (the best results are marked in bold font).

Methods	Classifiers	CLL_SUB_111	Breast	Lung	Tumors-11	SRBCT	GCM
*F*-test	*k*-NN	55.90 ± 6.73	56.54 ± 9.36	87.61 ± 2.51	56.37 ± 3.53	94.98 ± 1.52	52.09 ± 6.37
	RF	57.97 ± 6.34	58.09 ± 9.01	86.88 ± 1.72	55.07 ± 4.48	95.17 ± 2.37	50.27 ± 6.51
	SVM	57.07 ± 6.37	57.40 ± 8.17	85.38 ± 2.34	57.20 ± 4.14	95.37 ± 1.46	51.07 ± 6.81

RLR	*k*-NN	75.97 ± 6.24	61.63 ± 7.27	91.37 ± 2.52	82.37 ± 3.47	96.04 ± 1.61	63.96 ± 5.25
	RF	73.10 ± 5.31	61.87 ± 7.32	91.69 ± 2.07	82.77 ± 4.35	97.09 ± 1.72	60.73 ± 5.34
	SVM	74.63 ± 5.45	60.11 ± 7.29	93.34 ± 2.18	81.23 ± 4.07	97.08 ± 1.38	61.79 ± 5.29

WLMGS	*k*-NN	73.58 ± 5.37	59.37 ± 8.07	91.17 ± 2.47	79.18 ± 4.27	97.08 ± 2.92	59.79 ± 4.56
	RF	74.76 ± 6.37	61.13 ± 7.51	91.68 ± 2.17	82.53 ± 4.41	96.95 ± 1.60	59.75 ± 4.67
	SVM	74.99 ± 6.74	59.33 ± 7.24	92.26 ± 2.37	80.88 ± 4.38	97.24 ± 1.53	61.48 ± 4.35

LNNFW	*k*-NN	75.34 ± 6.73	60.17 ± 7.42	89.39 ± 2.74	81.00 ± 4.94	95.76 ± 1.19	61.44 ± 5.19
	RF	73.86 ± 5.42	59.82 ± 7.41	92.43 ± 2.22	81.75 ± 4.37	96.51 ± 2.53	61.38 ± 5.61
	SVM	74.69 ± 6.14	60.37 ± 7.51	91.74 ± 2.84	81.91 ± 4.07	97.62 ± 2.80	62.57 ± 5.33

GRSL-GS	*k*-NN	76.19 ± 5.72	63.94 ± 7.70	93.47 ± 2.72	82.14 ± 4.84	97.88 ± 1.34	64.14 ± 4.63
	RF	76.37 ± 5.43	62.94 ± 7.30	94.02 ± 2.67	82.12 ± 4.62	97.46 ± 1.20	62.70 ± 5.24
	SVM	75.76 ± 5.34	62.45 ± 7.74	93.09 ± 2.33	82.96 ± 3.77	97.66 ± 1.74	63.74 ± 4.31

AHEDL	*k*-NN	76.97 ± 5.44	65.34 ± 7.64	93.48 ± 2.13	84.74 ± 4.96	98.37 ± 1.15	65.34 ± 4.34
	RF	76.88 ± 5.19	64.18 ± 0.74	95.12 ± 2.64	82.79 ± 4.33	98.13 ± 1.15	64.24 ± 5.34
	SVM	76.37 ± 5.04	65.87 ± 7.32	94.15 ± 2.31	83.07 ± 3.46	98.61 ± 1.48	65.57 ± 4.64

G3CS	*k*-NN	78.37 ± 5.14	66.75 ± 7.34	94.78 ± 2.45	85.69 ± 4.32	98.87 ± 1.25	66.88 ± 4.64
	RF	77.19 ± 5.69	65.77 ± 0.31	94.39 ± 2.85	84.89 ± 4.36	98.42 ± 1.25	66.04 ± 5.37
	SVM	77.67 ± 5.81	66.96 ± 7.03	95.65 ± 2.18	84.88 ± 3.59	98.97 ± 1.38	67.11 ± 4.35

## Data Availability

The datasets used in this work are publicly available at: http://featureselection.asu.edu/datasets.php, http://portals.broadinstitute.org/cgi-bin/cancer/datasets.cgi.
